# Mitochondrial function as a therapeutic target in heart failure

**DOI:** 10.1038/nrcardio.2016.203

**Published:** 2016-12-22

**Authors:** David A. Brown, Justin B. Perry, Mitchell E. Allen, Hani N. Sabbah, Brian L. Stauffer, Saame Raza Shaikh, John G. F. Cleland, Wilson S. Colucci, Javed Butler, Adriaan A. Voors, Stefan D. Anker, Bertram Pitt, Burkert Pieske, Gerasimos Filippatos, Stephen J. Greene, Mihai Gheorghiade

**Affiliations:** 1 Department of Human Nutrition, Foods, and Exercise, Virginia Tech, 1035 Integrated Life Sciences Building, 1981 Kraft Drive, Blacksburg, Virginia 24060, USA; 2 Division of Cardiovascular Medicine, Department of Medicine, Henry Ford Hospital, 2799 West Grand Boulevard, Detroit, Michigan 48202, USA; 3 Division of Cardiology, Department of Medicine, University of Colorado Denver, 12700 East 19th Avenue, B139, Aurora, Colorado 80045, USA; 4 Department of Biochemistry and Molecular Biology, East Carolina Diabetes and Obesity Institute, Brody School of Medicine, East Carolina University, 115 Heart Drive, Greenville, North Carolina 27834, USA; 5 National Heart & Lung Institute, National Institute of Health Research Cardiovascular Biomedical Research Unit, Royal Brompton & Harefield Hospitals, Imperial College, London, UK; 6 Cardiovascular Medicine Section, Boston University School of Medicine and Boston Medical Center, 88 East Newton Street, C-8, Boston, Massachusetts 02118, USA; 7 Division of Cardiology, Health Sciences Center, T-16 Room 080, SUNY at Stony Brook, New York 11794, USA; 8 University of Groningen, Department of Cardiology, University Medical Center Groningen, Groningen 9713 GZ, Netherlands; 9 Department of Innovative Clinical Trials, University Medical Centre Göttingen (UMG), Robert-Koch-Straβe, D-37075, Göttingen, Germany; 10 University of Michigan School of Medicine, 1500 East Medical Center Drive, Ann Arbor, Michigan 48109, USA; 11 Department of Cardiology, Charité University Medicine, Campus Virchow Klinikum, and German Heart Center Berlin, Augustenburger Platz 1, 13353 Berlin, Germany; 12 National and Kopodistrian University of Athens, School of Medicine, Heart Failure Unit, Department of Cardiology, Athens University Hospital Attikon, Rimini 1, Athens 12462, Greece; 13 Division of Cardiology, Duke University Medical Center, 2301 Erwin Road Suite 7400, Durham, North Carolina 27705, USA; 14 Center for Cardiovascular Innovation, Northwestern University Feinberg School of Medicine, 201 East Huron, Galter 3-150, Chicago, Illinois 60611, USA

## Abstract

Heart failure is a pressing worldwide public-health problem with millions of patients having worsening heart failure. Despite all the available therapies, the condition carries a very poor prognosis. Existing therapies provide symptomatic and clinical benefit, but do not fully address molecular abnormalities that occur in cardiomyocytes. This shortcoming is particularly important given that most patients with heart failure have viable dysfunctional myocardium, in which an improvement or normalization of function might be possible. Although the pathophysiology of heart failure is complex, mitochondrial dysfunction seems to be an important target for therapy to improve cardiac function directly. Mitochondrial abnormalities include impaired mitochondrial electron transport chain activity, increased formation of reactive oxygen species, shifted metabolic substrate utilization, aberrant mitochondrial dynamics, and altered ion homeostasis. In this Consensus Statement, insights into the mechanisms of mitochondrial dysfunction in heart failure are presented, along with an overview of emerging treatments with the potential to improve the function of the failing heart by targeting mitochondria.

Heart failure (HF) is associated with substantial clinical burden and economic costs worldwide. The disease is particularly prevalent in elderly individuals, in whom the incidence and associated costs are projected to double over the next 20 years^1,2^. Economic costs associated with the management of patients with HF is estimated at >US$30 billion annually in the USA alone, and accounts for roughly 2–3% of total healthcare spending globally^3,4^. Despite these enormous costs, mortality from HF remains high. Death from HF within 5 years of diagnosis is common despite current optimal medical therapy. Mortality and rehospitalization within 60–90 days after discharge from hospital can be as high as 15% and 35%, respectively^5^. These event rates have largely not changed over the past 15 years, despite implementation of evidence-based therapy^5^. HF rehospitalization rates also remain high, with care typically focused on symptomatic relief. Patients with HF are often designated as having either reduced ejection fraction (HFrEF), or preserved ejection fraction (HFpEF). Patients with HFpEF also have poor prognosis after the first diagnosis^6^. Regardless of the HF aetiology, novel treatments that improve intrinsic cardiac function remain elusive.

Advances in the treatment of ischaemic and valvular heart disease have clearly improved patient survival. The residual cardiac dysfunction and associated comorbidities, however, have led, in the long-term, to the development of HF with attendant poor quality of life. Commonly prescribed HF medications, although beneficial in promoting some symptom relief, often do not fully address the underlying causes of progressive left ventricular dysfunction^7^. Most standard-of-care pharmacological approaches to HF act by reducing workload on the failing heart and, in doing so, attempt to rebalance energy supply and energy demand, albeit to a lower level (FIG. 1). Hallmarks of current therapies include modulation of neurohormonal abnormalities, unloading the heart (that is, vasodilatation), and/or reducing the heart rate — all important determinants of reducing myocardial oxygen consumption^8^. β-Blockers, ivabradine, and antagonism of the renin–angiotensin–aldosterone system all act in concert to reduce myocardial energy requirements and attenuate or prevent further adverse cardiac remodelling. Although these therapies have improved survival in patients with chronic ambulatory HFrEF over the past 2–3 decades, death and poor quality of life continue to adversely affect this ever-increasing patient population. This unmet need is probably not going to be met by drugs that modulate neurohormonal abnormalities and lower heart rates, because further intervention along these axes is likely to be counterproductive as hypotension and bradycardia become limiting factors. The search for more effective and complementary therapy for this patient population must be focused on improving the intrinsic function of the viable, but dysfunctional, cardiac unit — the cardiomyocytes^3,9^. The novel therapy must be haemodynamically neutral (no decrease in blood pressure or heart rate) and must target the myocardium as the centrepiece of the therapeutic intervention^10^.

The vast majority of phase III trials in patients with HF conducted in the past decade have been negative, arguably for the same reasons discussed above^11,12^. Furthermore, a relative underinvestment in cardiovascular drug development, as well as strategic abandonment by pharmaceutical companies of new therapies for which the risks are perceived to be higher than the rewards, have also contributed to slow development of drugs for HF^13^. Moreover, the development of effective therapies for HFpEF is imperative to treat this patient population, but the variability in HFpEF phenotypes (such as age, and the presence of diabetes mellitus or hypertension), and the difficulty in establishing reliable preclinical models of HFpEF, also hinder progress. Despite these obstacles, ample opportunity exists to improve HF treatments, provided the focus is directed towards cardiomyocytes and their intrinsic function.

A roundtable meeting was held in Stresa, Italy on 23 October 2015 to discuss the multifaceted problem of insufficient energy production in HF, and the role it has in progressive left ventricular dysfunction. This meeting was attended by academics, clinicians, and representatives from the pharmaceutical industry. The meeting focused on mitochondrial dysfunction as the source of energy deprivation in HF, and how correction of mitochondrial dysfunction using emerging novel therapies might lead to functional improvement of the HF phenotype. This Consensus Statement summarizes the findings from that roundtable discussion.

## Bioenergetics of the beating heart

**Figure 1:**
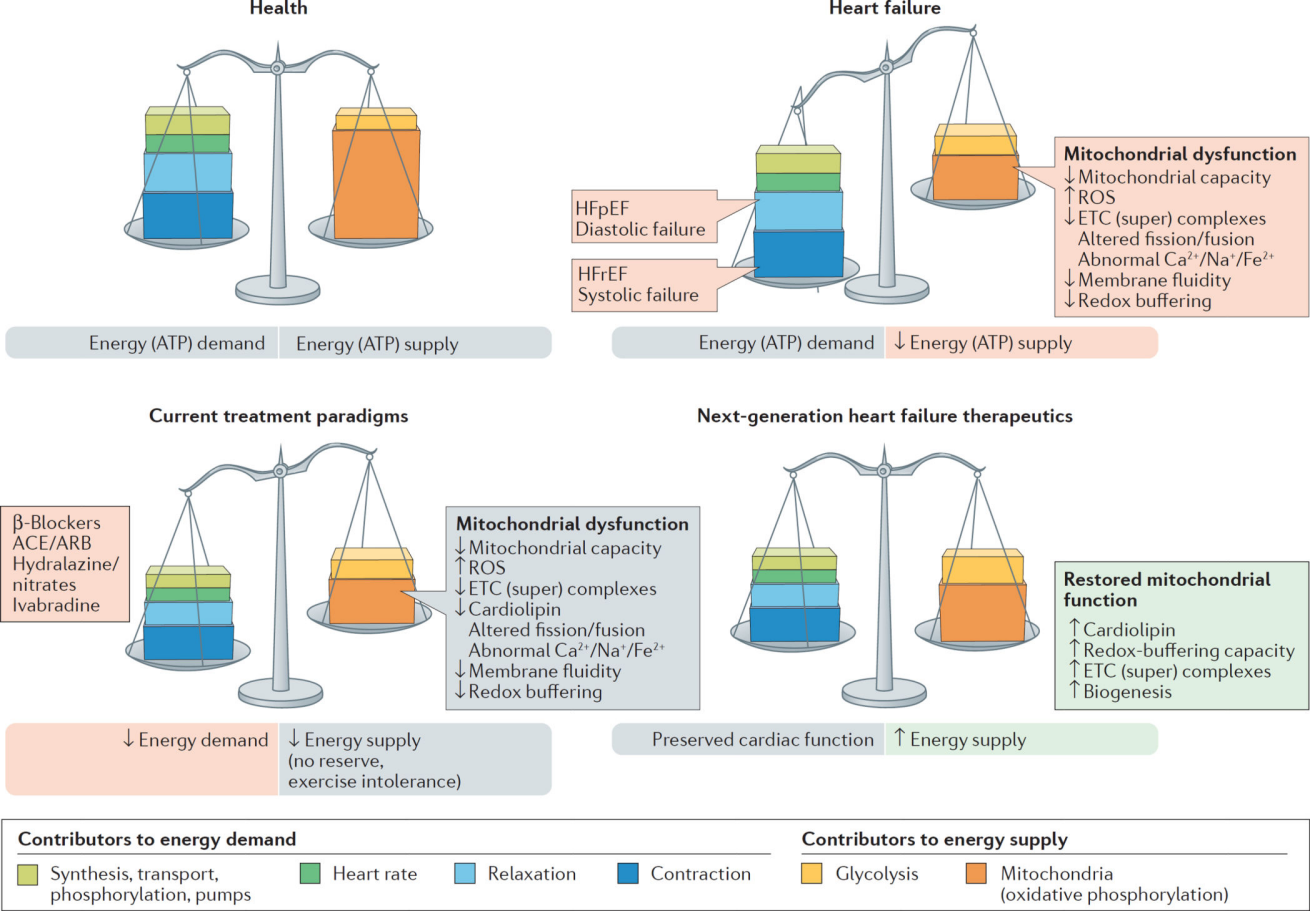
Energy supply–demand matching in health and heart failure The delicate balance between cardiac demands for energy and supply of energy is tipped in heart failure, in which energy supply cannot match demand. Next-generation therapeutics can improve on existing standard-of-care therapies by bolstering mitochondrial energy production. ACE, angiotensin-converting enzyme; ARB, angiotensin II-receptor blocker; ETC, electron transport chain; HFpEF, heart failure with preserved ejection fraction; HFrEF, heart failure with reduced ejection fraction; ROS, reactive oxygen species.

Aristotle considered the heart to be the body’s furnace, radiating energy in the form of heat^14^. Given the astounding energetic cost of cardiac function, this concept is not so far from the truth. Humans produce and consume roughly their body weight in ATP (about 65 kg) every single day^15^. The heart accounts for only ~0.5% of body weight, but is responsible for roughly 8% of ATP consumption. This high energy flux is dynamic: the heart stores only enough energy to support pumping for a few heart beats, turning over the entire metabolite pool approximately every 10 s even at resting heart rates^16^. As the most metabolically active organ in the body, the heart possesses the highest content of mitochondria of any tissue. Mitochondria comprise 25–30% of cell volume across mammalian species^17,18^, with only the myofilaments being more densely packed within cardiac myocytes. The high mitochondrial content of cardiomyocytes is needed to meet the enormous energy requirement for contraction and relaxation (which is also an active process). About 90% of cellular ATP is utilized to support the contraction–relaxation cycle within the myocardium^19^. ATP-dependent release of actin from myosin is required for both contraction (as myosin heads cycle through cross-bridges with actin) and relaxation. Cellular sequestration of calcium back into the sarcoplasmic reticulum during diastole also requires a tremendous amount of ATP. Cells sustain the energy requirements necessary to support cardiac function through remarkable metabolic supply–demand matching^20,21^ (FIG. 1). Bioenergetic homeostasis is accomplished almost exclusively through an ‘energy grid’ comprised of a mitochondrial network and their associated phosphate- transfer couples. Cardiac mitochondria must operate at high efficiency levels to respond instantaneously to the energetic needs of contractile units, a demand that is ever-changing and necessitated by the body’s dynamic requirements for oxygen-bearing blood.

Myocardial energy requirements are more pronounced during physical activity, when demands for energy increase to maintain cardiac function commensurate with the needs of the body. However, other mitochondrial abnormalities besides energy deprivation during physical activity can contribute to the pathologies seen in patients with HF. Mitochondrial abnormalities in HF are not only a question of reduced capacity to generate ATP (even though that capacity is reduced at rest in HF compared with resting normal), but can also be directly linked to cardiomyocyte injury and death and, therefore, to disease progression. Abnormal mitochondria are a major source of reactive oxygen species (ROS) production, which can induce cellular damage. Abnormal mitochondria can promote programmed cell death through the release of cytochrome *c* into the cytosolic compartment and activation of caspases. Therefore, mitochondria directly influence ongoing cell injury and death. Mitochondrial abnormalities have also been implicated in aberrant cellular calcium homeostasis, vascular smooth muscle pathology, myofibrillar disruption, and altered cell differentiation, all important issues in cardiovascular disease, including HF.

## Mitochondria in cardiomyocytes

Mitochondria are primarily located within subsarcolemmal, perinuclear, and intrafibrillar regions of the cardiomyocyte. Although they are symbiotic partners with the other cellular compartments, mitochondria are in many ways discrete entities. Mitochondrial dynamics in the form of fission, fusion, and autophagy are highly regulated processes that are essential for energy production and structural integrity of the organelles^22–29^. Altered mitochondrial biogenesis, fragmentation, and hyperplasia have been observed in studies of human^30^ and animal models^31,32^ of HF. These effects seem to be caused by altered expression of proteins that regulate mitochondrial dynamics^33^. As many of these factors are ‘master regulators’ of mitochondrial metabolism, these changes might be directly related to the decreased capacity to oxidize fatty acid substrates often seen in HF^34,35^.

Mitochondria have their own DNA (mtDNA) and a genetic code that is distinct from the host-cell nuclear DNA. mtDNA is circular in shape, analogous to DNA found in lower organisms, and a primitive fingerprint leftover from bacterial origin. Evolutionary selection pressures have led to mitochondria ‘outsourcing’ almost all their protein-making needs to their cellular hosts. The overwhelming majority (>99%) of mitochondrial proteins come from nuclear-encoded DNA. These proteins are synthesized via cellular protein synthesis machinery, and are actively imported into mitochondria through mitochondrial membrane transporters^36^. mtDNA encodes 13 protein subunits found within three of the electron transport protein complexes, and a handful of ribosomal and transfer RNAs^37^. These proteins are made in specialized ribosomes or ‘mitoribosomes’, which are physically attached to the mitochondrial inner membrane^38^.

Many inherited familial cardiomyopathies (both adult and paediatric) are associated with mtDNA mutations^39^. In humans, mitochondria are maternally inherited^40^, owing to high mitochondrial density in the egg and the active degradation of mitochondria in the sperm during fertilization^41^. The proximity of mtDNA to sites of mitochondrial ROS generation, poor repair mechanisms, and a lack of protective histones combine to make mtDNA particularly susceptible to oxidative injury and mutation.

Mitochondrial genetics contribute to cardiomyopathies by expressing mutant proteins that influence energy homeostasis. With 1,000–10,000 genes per mitochondria (polyploidy), mitochondrial genetics operate on population-based (instead of Mendelian) principles^37^. Mutated mtDNA is found alongside nonmutated copies, leading to mitochondrial ‘heteroplasmy’. The extent of heteroplasmy in mutated mtDNA influences the susceptibility to inherited mitochondrial disease^42^. Mutated mtDNA can be found in 1 in 200 individuals, a frequency that is 20-fold higher than the incidence of mitochondrial disease. This mismatch indicates that healthy individuals often harbour mutated mtDNA that has no observable phenotypic consequences until a certain mutation threshold is reached^37^. Although very early in preclinical development, various innovative approaches to reduce the extent of heteroplasmy using genome editing might ultimately lead to effective therapy for HF caused by genetic mitochondrial disease^43–45^. Given that mitochondrial abnormalities, such as increased ROS production, altered mitochondrial energetics, and impaired mitochondrial ion homeostasis, are observed in genetic mitochondrial diseases as well as HF, innovative approaches that target mitochondrial dysfunction might share efficacy across these diseases.

## Heart failure is a bioenergetic disease

**Figure 2:**
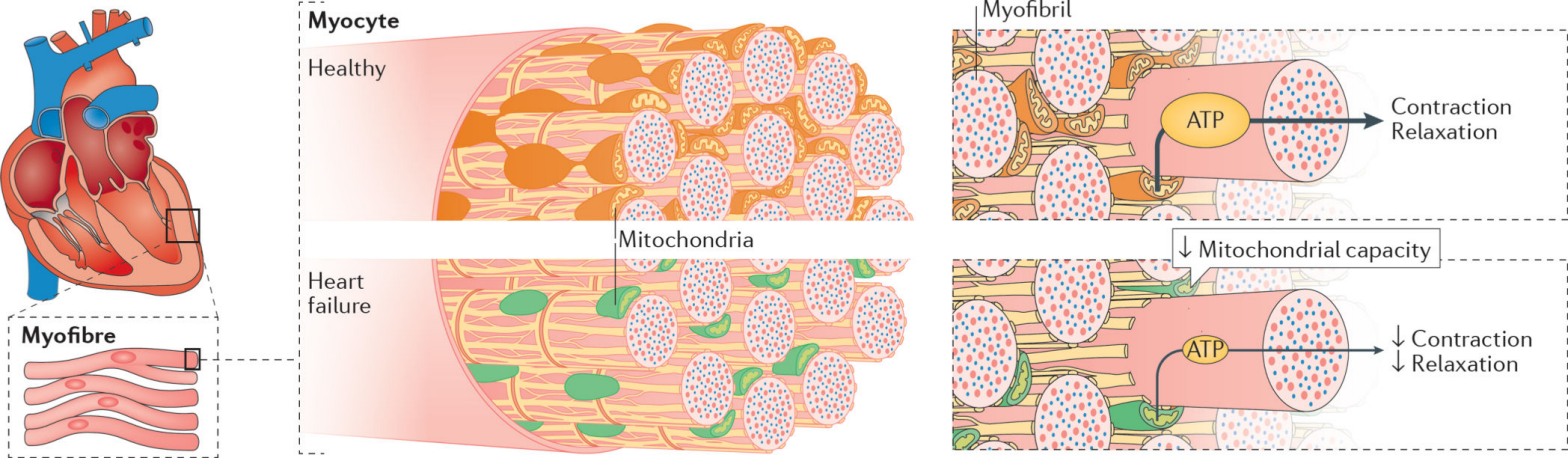
Impaired mitochondrial capacity and function in heart failure Decreased capacity of mitochondria to generate and transfer energy within heart cells results in energy deficits, which influences all cellular processes that require energy, most notably the processes of contraction and relaxation.

The ‘myocardial power grid’ consists of mitochondrial ATP supply that transfers energy throughout the cell along intracellular phosphotransfer buffering systems (FIG. 2). Mitochondria utilize carbon sources from food substrates, which are catabolized and passed through the Krebs cycle and are then channelled through a series of redox reactions along the inner mitochondrial membrane. The oxidation of these substrates creates a proton electrochemical gradient, predominantly in the form of mitochondrial membrane potential (ΔΨ_m_)^46^. Protons that reenter the mitochondrial matrix through complex V (mitochondrial ATP synthase) liberate energy that phosphorylates ADP, regenerating ATP. Newly synthesized ATP is rapidly transferred out of mitochondria and energy is subsequently distributed throughout the cell via reversible phosphate exchange networks, primarily catalysed by creatine kinase and adenylate kinase-associated reactions^16,47^.

The evidence that HF involves impaired cellular energy production and transfer is considerable (TABLE 1). Among studies that have directly examined energetics in human HF, all but three noted some form of bioenergetic impairment in the failing heart. This decrement in bioenergetics is reflected by a decrease in cellular ATP, phosphocreatine (PCr), or the PCr/ATP ratio. Impaired bioenergetics affect patients with HFrEF and those with HFpEF (TABLE 1).

**Figure 3:**
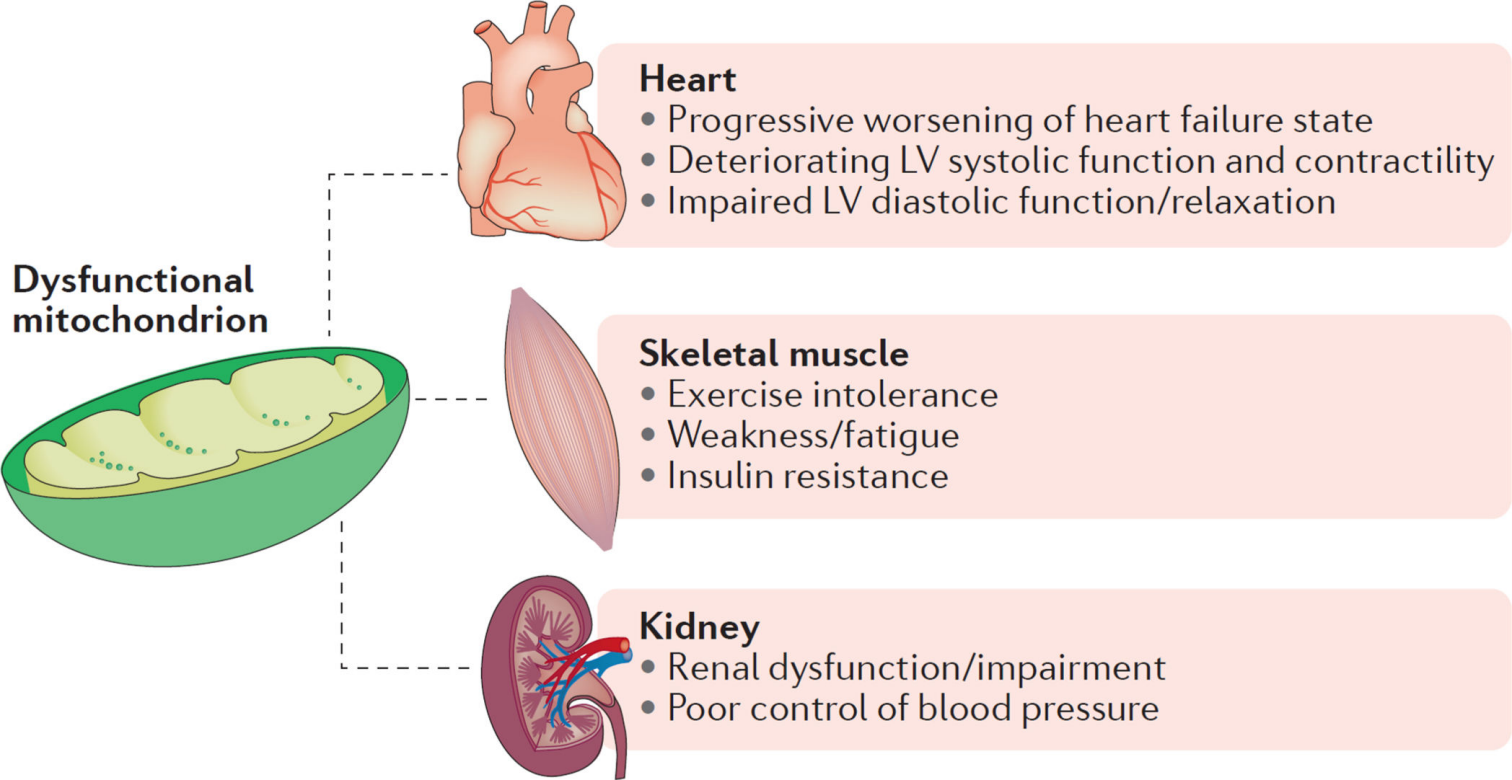
Mitochondrial contribution across multifaceted symptoms of heart failure Aberrant mitochondrial energy production is involved in many symptoms commonly found in patients with heart failure, including skeletal muscle dysfunction and renal pathologies. LV, left ventricular.

Although it is difficult to tell from the heterogeneous patient population included in TABLE 1, the progression to HF is likely to be associated with a gradual decline in bioenergetic reserve capacity that ultimately reaches a critical threshold, after which endogenous mechanisms can no longer compensate for faltering energy supply^48^. Attempts to improve bioenergetics in HF tend to focus on mitochondrial energy production as a target, because direct augmentation of myocardial creatine with oral creatine supplementation is thwarted by a decreased capacity to transport creatine into the failing cardiomyocytes^49^. Skeletal muscles also show mitochondrial dysfunction in HF, contributing to the exercise intolerance that characterizes the HF state^50^. Abnormal mitochondrial function has also been reported in patients with renal insufficiency^51^, and in patients with insulin resistance^52^. Given that patients with HF often manifest both renal insufficiency and insulin resistance, treating mitochondrial dysfunction in HF derives benefits that go beyond improving cardiac function (FIG. 3).

Several interventions are currently being tested in clinical trials to stimulate mitochondrial biogenesis in HF. These include epicatechin and resveratrol, which are naturally-occurring polyphenols found in foods such as red wine, green tea, and dark chocolate. Preclinical HF models suggest that these molecules are biologically active^53–55^, and some success in improving cardiac function has been reported in small trials of patients with myocardial infarction^56^. Larger trials in patients with HF are required.

## Mitochondrial substrate selectivity

Substrate utilization in the failing heart has been extensively reviewed previously^57–60^. Overall, altered substrate metabolism seems to be centrally involved in HF, although the direction of the metabolic alterations is complex and is likely to depend on the particular stage of HF progression and differences in the availability of substrate (whether the heart is in a ‘fed’ or ‘fasted’ state)^58,59^.

The heart utilizes different substrates simultaneously to produce energy. Mitochondrial fatty acid oxidation (FAO) is the predominate substrate used in the healthy adult human heart, being responsible for 60–80% of cardiac ATP production, followed by lesser contributions from glucose, lactate, and ketone bodies^61^. However, the heart can shift the relative contribution of these substrates in an effort to adapt to varying physiological conditions. Under conditions of low oxygen content, such as ischaemia and HF, ATP content is thought to decrease by as much as 40%^3^. In HF, fatty acid oxidation and the oxidative capacity of the mitochondria decline, and can no longer maintain sufficient levels of ATP, especially during conditions of increased cardiac workload such as exercise. The failing heart shifts its predominant fuel source from mitochondrial FAO toward glycolytic pathways. This switch is most apparent in late and end-stage HF^57^, and is 30% more energetically efficient in the failing heart, because more ATP is produced per mole of oxygen during carbohydrate oxidation^62^. Numerous studies investigating FAO, glucose oxidation, and (to a lesser extent) ketone body oxidation have aimed to establish a metabolic phenotype, underlying molecular mechanisms, and potential therapeutic targets of the failing heart.

The reduction in fatty acid uptake and FAO that occurs during HF might be owing to dysregulated molecular mechanisms responsible for fatty acid metabolism. For example, the level of peroxisome proliferator-activated receptor-α (PPARα), a transcription factor highly expressed in the heart and responsible for fatty acid transport into the mitochondria and peroxisomes, has been reported to be downregulated in both animal models and humans with HF^63,64^. Similarly, tissue from animals and humans with HF has reduced activity of the transcription factor responsible for mitochondrial biogenesis, PPAR-γ co-activator (PGC)-1α^64,65^. Because these transcription factors have a critical role in the regulation of cardiac mitochondrial energy production, these data suggest that decreased PPARα and PGC-1α activity might be an important precursor leading to impaired FAO during HF. Therefore, further inhibition of FAO to increase glycolytic flux via PPARα and/or PGC-1α is a plausible therapeutic target. Small-molecule regulators of PGC-1α are needed, and animal models overexpressing the transcription factor are inherently problematic, ostensibly owing to increased mitochondrial biogenesis-induced cardiomyopathy^66^. Similarly, PPARα antagonists in animal models of HF have yielded inconclusive data^67^, whereas clinical PPARα ligands are reportedly safe, but their efficacy in a HF population is currently unknown^61^. Although the safety of PPARα ligands is promising, further evidence demonstrating their efficacy in both animal models and humans with HF is needed.

Levels of circulating free fatty acids might be higher in the failing heart than under healthy conditions owing to hormonal stimulation. The rise in serum catecholamine levels increases plasma free fatty acid concentrations, and subsequently stimulates FAO^68^. As a result, reducing the availability of circulating free fatty acids via transient adrenergic antagonists might be a viable therapy to inhibit FAO and increase glycolytic ATP production. Traditionally, β-adrenergic receptor antagonists are used in HF owing to their negative ionotropic effects that reduce cardiac workload and spare oxygen by decreasing sympathetic activity^68^. Many, such as carvedilol, have been clinically shown to lessen infarct size after ischaemia by decreasing sympathetic activity, followed by inhibition of mitochondrial fatty acid uptake and increased glucose oxidation^69^.

Malonyl-CoA endogenously regulates fatty acid concentrations by controlling the activity of carnitine *O*-palmitoyltransferase (CPT) 1, a rate-limiting enzyme in mitochondrial fatty acid uptake^68^. When intracellular levels of malonyl-CoA are increased, CPT1 is inhibited and mitochondrial fatty acid uptake is stopped^70^. The intracellular concentration of malonyl-CoA is dependent on the balance between its synthesis via acetyl-CoA carboxylase and degradation via malonyl-CoA decarboxylase. Therefore, the upregulation of acetyl-CoA carboxylase or inhibition of malonyl-CoA decarboxylase would increase intracellular malonyl-CoA levels, and prevent mitochondrial uptake of free fatty acids to reduce FAO. As expected, inhibiting malonyl-CoA decarboxylase in animal models has reportedly improved cardiac function after ischaemia, reduced cardiac FAO, and increased glycolytic flux^71,72^. Studies of malonyl-CoA decarboxylase inhibitors in patients with HF are needed.

Trends in glucose oxidation across the spectrum of HF are more variable, particularly among animal models of HF^58^. Compensatory substrate switching towards glucose use has been observed in both animal models and humans^59^, with a higher contribution coming from glycolysis. Stimulating mitochondrial glucose oxidation, either directly or by inhibiting fatty acid catabolism, has been suggested as a viable therapeutic strategy to compensate for the energetically ‘starved’ failing heart^59^.

Ketone body metabolism also seems to be altered in HF. Ketones are formed in the liver via fatty acid metabolism, and provide a small substrate pool for oxidation within the myocardium. In conditions such as diabetes or starvation, ketone catabolism is upregulated in response to lowered insulin availability and higher fatty acid levels^57,73^. Studies have reported increased ketone utilization in the severely failing heart in humans^73,74^. Further research is needed to understand the role of ketone oxidation in the failing myocardium, and to determine whether targeting ketone metabolism is a plausible therapy to improve energetics in HF.

Novel insights into the regulation of metabolic substrate demand in the heart have been provided through studies of microRNAs and acetylation of mitochondrial lysine residues. Alterations in microRNA levels through any number of upregulation and downregulation events can alter substrate utilization in the heart^75^. Alterations in protein levels modulated by microRNA expression have been proposed to have important implications for glycolysis, β-oxidation, ketone metabolism, the Krebs cycle, and the electron transport chain (ETC)^75^. For example, increased levels of ROS can alter calcium handling in HF by modifying microRNA that leads to inhibition of sarcoplasmic/endoplasmic reticulum calcium ATPase (SERCA) 2a transcription^75^. Post-translational modification via lysine acetylation has been suggested to have an important role in metabolic enzyme regulation in the mitochondria^59^.

## Overactivation of the SNS

As all substrates converge on mitochondria, understanding the specific abnormalities that occur in HF is central to the development of new treatments. ROS production increases in many aetiologies of HF, a phenomenon that might be directly related to increased sympathetic nervous system (SNS) tone^76^. Sustained sympathetic drive and chronically elevated circulating catecholamines — processes that are normally transient to mediate acute increases in cardiac output — are commonly observed in patients with HF (particularly HFrEF)^77,78^. Chronic stimulation of β-adrenergic receptors has been directly linked to mitochondrial ROS production through adrenergic receptor-mediated second messenger signalling^79^,^80^. ROS-mediated initiation of mitochondria-dependent cell death cascades has been repeatedly observed after chronic sympathetic activation, leading to overall declines in mitochondrial function^81–86^. These processes can be amplified by the formation of aminochromes, catecholamine metabolites known to impair mitochondrial redox balance^87^. Attenuation of HF pathology with β-blockers and rennin–ngiotensin–aldosterone antagonism has resulted in substantial clinical improvements^88^, and is likely to relieve some of the mitochondrial dysfunction that accompanies increased sympathetic tone. The capacity to complement these existing background therapies with compounds that directly target mitochondrial dysfunction is a potentially promising novel paradigm (FIG. 1).

## Increased ROS production

**Figure 4:**
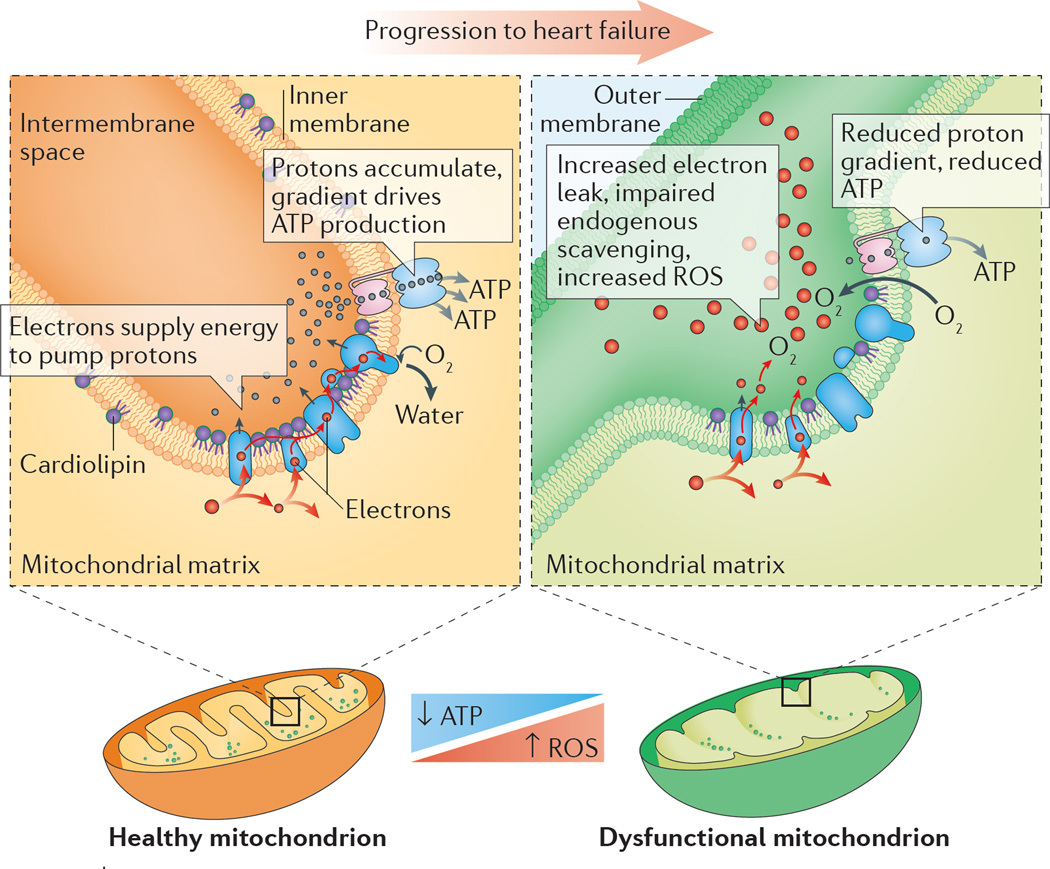
Impaired mitochondrial energy production along the inner membrane Enzyme complexes responsible for energy production are packed into the mitochondrial inner membrane, often with the help of phospholipids such as cardiolipin. Failing mitochondria often display altered morphology, decreased ATP-generating capacity, heightened production of reactive oxygen species (ROS), abnormal cardiolipin levels, and impaired supercomplexes.

Cellular ROS production occurs when ROS formation outpaces or exhausts compensatory signals and overwhelms endogenous scavenging systems^89–91^. ROS are produced at several different sites within cells, both within and outside of mitochondria (reviewed in detail previously^92–95^). Mitochondrial ROS production occurs at various sites along the inner mitochondrial membrane as well as in the mitochondrial matrix by components of the ETC and the Krebs cycle, respectively^96^ (FIG. 4). ROS production is typically low under normal physiological conditions^93^, and is kept in check by intracellular and intramitochondrial scavenging systems. Pathological ROS levels in the heart typically occur when ROS production outpaces endogenous scavenging capacity. ROS (and other associated reactive intermediates) can damage proteins and lipids, trigger cell-death cascades, and evoke synchronized collapses in the cellular energy grid^97^,^98^. Heightened mitochondrial ROS production and downstream ROS-mediated damage has been reported in patients with HF as well as in preclinical models of the disease^31,99–101^.

Although ROS are typically associated with pathological states, ROS levels in the heart *per se* are best characterized by the term ‘hormesis’: small amounts can evoke adaptive signalling and create beneficial, compensatory responses. Modest production of ROS has been shown to mediate beneficial myocardial signalling involved in physiological responses such as (transient) sympathetic drive^102^, many preconditioning paradigms^103^, cardiac mitochondrial quality control^104^, and exercise^105^. Exercise training is known to augment endogenous ROS-scavenging mechanisms in the heart^105–107^, restore bio-energetic efficiency in porcine models of HFpEF^108^, and improve symptoms and quality of life in trials involving patients with HFrEF^109,110^ or HFpEF^111^. Consistent with the ROS hormesis concept, several studies have noted that administration of high doses of ROS scavengers can abolish the beneficial effects of exercise^112^,^113^, including humans taking oral vitamin C or E supplements^114^.

Mitochondrial production of ROS depends on the mitochondrial membrane potential. Increased expression of mitochondrial uncoupling proteins in HF^115^ might be a compensatory mechanism to reduce ROS by ‘uncoupling to survive’^116^, whereby a reduction in mitochondrial membrane potential is postulated to lower ROS emission from mitochondria. This view is popular and almost dogmatic, but the decrease in ROS production by uncoupling is a prominent effect during mitochondrial state 4 respiration (no ADP). Heart mitochondria, however, are never respiring in state 4. Pathological ROS production in cardio-myocytes is likely to be more closely linked to decreased or collapsed membrane potential and/or depletion of the NADPH pool^117–119^, whereby ROS production overwhelms endogenous scavenging through mitochondrial membrane-dependent mechanisms^89^.

The repeated lack of benefits of ROS scavenging compounds in clinical trials of patients with HF^11,120,121^ continues to plague cardiovascular drug development, suggesting that oxygen radical scavenging *per se* is not a plausible mechanism of action for long-term improvements in HF. Lack of tissue permeability, poor intra-cellular targeting, and ineffective therapeutic doses might contribute to the poor translation of benefits of anti oxidants to date. This approach to therapy, however, might ultimately succeed when novel scavenging compounds that overcome permeability and targeting problems, such as XJB-5–131 (REFS 122,123), mitoTEMPO^124,125^, and EUK8/EUK134 (REFS 126–128), are tested in humans.

## Abnormalities of mitochondrial ETC

Decrements in individual electron transport complexes, particularly complex I and/or IV activity, have been observed in animal models^129^ and humans^35^ with HF. Electron transport system proteins seem to aggregate into functional supercomplexes^130–132^, and a loss of mitochondrial supercomplexes, which is postulated to have a causal role in mitochondrial ROS generation^133^, has been noted in HF^134^.

Several approaches are being developed to improve the efficiency of the ETC in HF. The coenzyme Q (ubiquinol/ubiquinone CoQ) pool comprises a redox-cycling coenzyme found in the ETC. CoQ is typically synthesized *de novo* and undergoes a two-electron reduction from substrates fed into complexes I and II, and is then oxidized as it donates electrons into complex III. As a redox cycler, the ubiquinol/ubiquinone couple can both accept and donate electrons, depending on the redox potential^135^. Incomplete, one-electron reduction of CoQ produces semiquinone, itself a highly reactive radical. A reduced CoQ pool could potentially feed electrons ‘backwards’ towards complex I, which results in reverse electron transfer and ROS generation^136^. Decreased circulating CoQ has been observed in patients with HF^137,138^, with an inverse correlation observed between plasma CoQ and mortality^139^. In the Q-SYMBIO trial^140^, the efficacy of CoQ was tested in a small (*n* = 420), double-blind, placebo- controlled study in patients with HF and showed a reduction in mortality after 2 years of treatment. Although the Q-SYMBIO trial was fairly small, the promising findings triggered interest in the development of other CoQ analogues that more effectively target mitochondria. New quinone conjugates that are tethered to lipophilic, cationic triphenylphosphonium moieties, such as MitoQ, SkQ, and other plastoquinones, might improve the delivery of CoQ to mitochondria^141–143^, and have shown some promise in preclinical models of HF^144^. A potential problem with the use of these compounds is that they are self-limiting, in that they can depolarize mitochondria and inhibit mitochondrial respiration at high concentrations^145^. Several short-chain synthetic CoQ analogues are also in development, including EPI-743 (REF. 146) and idebenone^147^. These compounds have shown promise in small trials of genetic mitochondrial disease^148,149^, but have not yet been tested in larger trials of human HF.

Aberrant mitochondrial membrane phospholipids in HF are integrally involved in ETC dysfunction. A membrane phospholipid integral to optimal function of the ETC and whose content and composition are altered in HF is cardiolipin. Cardiolipin resides in the inner mitochondrial membrane (FIG. 4) and, unlike most phospholipids that have two acyl tails, cardiolipin has four acyl chains. In mammalian hearts, these chains are enriched with linoleic acid (18:2)_4_. Cardiolipin decrements are observed in both paediatric^150^ and adult^151,152^ patients with HF. Cardiolipin is essential for the activity of ETC complexes, membrane transporters, mitochondrial ion homeostasis, and ROS production^153^. Given that most mitochondrial complexes associated with energy production are oligomers composed of many subunits, cardiolipin is proposed to act as molecular ‘glue’ holding these subunits together^154–156^. Approaches that target cardiolipin are likely to improve electron transport across the ETC and, in doing so, might be beneficial in treating HF.

A compound that targets cardiolipin in the mitochondria that is currently in clinical development is the cell-permeable peptide MTP-131 (also called elamipretide or Bendavia). An analogue of MTP-131 (SS-31) was serendipitously discovered by Szeto and Schiller in attempts to identify small peptides with opioid-receptor binding properties^157^. MTP-131 has no discernible opioid-receptor activity^158^, but was found to localize to the inner mitochondrial membrane^159^, reduce myocardial ischaemia– reperfusion injury^112,160,161^, improve renal function^51,162^, and restore skeletal muscle function^163^. MTP-131 is not a direct ROS scavenger^164^, and is postulated to act by interacting with cardiolipin^165^ to interrupt the vicious cycle of ROS-mediated cardiolipin oxidation and subsequent loss of energetics^119,166^. MTP-131-mediated improvements in mitochondrial energetics have been observed across a number of different tissues in animal models of disease, including the myocardium^161,163,164^. Of note, MTP-131 can improve mitochondrial bioenergetics by improving respiratory supercomplex formation (D. A. Brown, unpublished work).

MTP-131 is currently being investigated in several phase II clinical trials. Preclinical studies in mouse models of HF have demonstrated efficacy using MTP-131. In a mouse model of HF induced by aortic constriction, MTP-131 improved left ventricular function, reduced hypertrophic remodelling, and restored mitochondrial function^167^. In complementary studies, MTP-131 administration substantially reduced maladaptive remodelling, preserved cardiac function, lowered β-adrenergic- mediated calcium overload, and restored mitochondrial protein expression^168–170^. A substantial improvement in cardiac function with MTP-131 has been demonstrated in a porcine model of HFpEF^171^ and a canine model of HFrEF^172^. Beneficial improvements in ejection fraction were associated with improved activity or expression of mitochondrial complexes I, IV, and V, and a normalization of cardiolipin levels^172^. As the HF syndrome influences many different tissues (FIG. 3), the evidence that MTP-131 also improves skeletal muscle function, exercise capacity, and renal function adds to the promise of this emerging therapy^51,163,173,174^.

## Blockers of the MPTP

The mitochondrial permeability transition pore (MPTP) is a nonspecific pore that opens in response to increased calcium levels and oxidative challenge, and is associated with ROS production, apoptotic cell death, and mitochondrial dysfunction. Increased proclivity of MPTP opening occurs in both acute and chronic heart disease, and numerous preclinical studies have demonstrated efficacy in cardiac pathology with MPTP blockers, such as cyclosporin, NIM811, and TRO40303 (reviewed previously^175–179^). Although the opening of the MPTP has historically been thought of as a pathological event leading to cell death, studies now suggest that transient MPTP opening might be a physiological ‘reset’ mechanism to prevent mitochondrial calcium overload. Rare, transient openings of the MPTP have been observed in individual mitochondria of primary cardiomyocytes^180^. Small, brief MPTP openings were found to be more frequent in HF cardiomyocytes, and were associated with transient mitochondrial depolarization and mitochondrial calcium release. If opening of these pores might be a normal compensatory mechanism akin to ‘pressure release valves’, the concept of treating HF by blocking them becomes increasingly difficult. Ongoing uncertainty regarding the molecular identity of the MPTP further complicates the development of novel therapies that act on the pore^176,181–185^. The MPTP seems to be comprised of ATP synthase (complex V) dimers and to be gated by mitochondrial matrix calcium content via cyclophillin D^186,187^.

Clinical studies have failed to demonstrate efficacy in most^188,189^, but not all^190,191^, studies; however, most of these studies focused on reducing acute cardiac ischaemia– reperfusion injury and not in limiting left ventricular dysfunction in HF. Chronic administration of cyclosporin has been linked with renal pathology and immunosuppresfsion^192,193^, and cyclosporin was found to evoke systemic hypertension in porcine models of HFpEF^194^. Accordingly, cyclosporin is not an appropriate approach for the long-term management of HF. Further work with alternative MPTP blockers is needed to determine whether inhibiting or delaying MPTP opening is a clinically plausible approach to alter the progression of HF.

## Cellular/mitochondrial ion homeostasis

Aberrant handling of several different ions within the mitochondria has been observed, mostly in animal models of HF. Heightened levels of free iron can increase ROS through Fenton chemistry. Changes in cellular iron handling have been noted in HF^7,195^, and orally-available iron chelators such as deferiprone seem to redistribute iron from tissues, including the mitochondrial space, into the circulation^196^. Although a potential exists to treat HF by chelating cellular iron, no study to date has shown functional improvements of the failing heart, although several clinical trials are currently underway.

Impaired cellular calcium handling that leads to decrements in excitation–contraction coupling is noted across HF aetiologies, and contributes to poor cardiac mechanics and to arrhythmogenesis^197–200^. Mitochondria can directly influence cellular calcium dynamics, because many of the membrane-bound pumps required for cytosolic calcium release and removal are energy- dependent and ROS-dependent. Altered calcium handling has been implicated in HFpEF, in which abnormal calcium dynamics impair relaxation. Short-term administration of ivabradine to slow the heart rate led to modest benefits in patients with HFpEF, ostensibly by providing more time for calcium- dependent relaxation^201^. The vast majority of calcium resequestration into the sarcoplasmic reticulum, obligatory for diastolic relaxation, occurs through SERCA2a, which has been shown to be downregulated in HF^202–204^. Overexpressing SERCA2a has shown promise in animal models of HF^205,206^, although several barriers (such as the development of neutralizing antibodies) still exist before gene transfer realizes its full translational potential^207^. Furthermore, increased ROS can oxidize proteins associated with the ryanodine receptor calcium-release channel, which can lead to calcium leaking out of the sarcoplasmic reticulum during diastole^208^. Increased intracellular sodium levels in HF^209–212^ also contribute to poor calcium handling through mechanisms involving sodium–calcium exchange. Given that calcium is central to maintaining bioenergetic supply–demand matching^21,213^, sodium overload alters cellular and mitochondrial calcium fluxes and impairs bioenergetic supply– demand matching in HF^214^. Although very early in development, inhibitors of the mitochondrial sodium– calcium– (lithium) exchanger^215^, such as CGP-37157, have been shown to improve cardiac function in preclinical models of HF^216,217^. Inhibiting the sarcolemmal sodium– calcium exchanger might also be a promising approach, as demonstrated in a preclinical model of HFpEF^218^.

Another compound in clinical development to improve cardiac efficiency in HF is omecamtiv mecarbil (CK-1827452). This drug increases the calcium sensitivity of the myofilaments^219^, which prolongs the duration of systole in animal models and in human HF^220–222^. Two substantial phase IIb, double-blind, randomized studies comparing omecamtiv mecarbil and placebo have been conducted. In the ATOMIC-HF trial^223^, omecamtiv mecarbil was administered for 48 h intravenously to patients with acute HF. Overall, the study was neutral (with some evidence of a symptomatic benefit at higher doses), but suggested omecamtiv mecarbil was safe. In the COSMIC-HF trial^224^, an oral formulation of omecamtiv mecarbil was associated with improvements in cardiac function over 20 weeks, with an effect that persisted for 4 weeks after stopping the drug, suggesting that improved function had produced favourable structural remodelling. Despite the promise of omecamtiv mecarbil, concerns about elevated levels of serum troponin^225^, metabolic inefficiency^226^, and impaired cardiac relaxation^227^ must be assuaged by larger clinical trials to understand fully whether this approach can improve prognosis in HF.

## Conclusions

The vast majority of HF trials over the past decade have been neutral, and event rates remain unacceptably high. Perhaps most alarming, no proven therapies exist for patients with worsening chronic HF or HFpEF — populations that collectively comprise the majority of the total HF population. Moreover, although systemic blockade of maladaptive neurohormonal responses has improved outcomes in HFrEF, these agents also lower blood pressure and/or heart rate, and development of new haemodynamically active drugs for stepwise addition to existing therapies raises safety and tolerability concerns. Therefore, an ideal novel therapy would be haemodynamically neutral and target the myocardium as the centrepiece of the therapeutic mechanism. In this context, overwhelming evidence from both preclinical and clinical studies indicates bioenergetic insufficiency in HF. Studies using preclinical models of the disease continue to advance our understanding of the cellular and molecular mechanisms that contribute to poor bioenergetics of the failing heart. Considerable potential exists to fill this unmet need, mitigate the economic burdens, and reduce symptoms in patients with HF by focusing on the development of new therapeutic modalities that target mitochondrial abnormalities in HF.

## Figures and Tables

**Table 1:** Bioenergetic changes in human heart failure

Patient characteristics (*n*)	ATP	PCr	PCr/ATP	Notes
NYHA class II (29), class III (8)^228^	NR	NR	↓	Decrease in PCr/ATP ratio in patients with HFpEF
NYHA class I (10), class III (8), class IV (1)^229^	NR	NR	↓	Decrease in PCr/ATP ratio in HCM correlated with presence of fibrotic areas in myocardium of left ventricle
LVH (20); LVH and CHF (10); no LVH (10)^230^	↓	↓	↓	Decrease in ATP flux through CK; 30% decrease in PCr/ATP ratio
NYHA class I (1), class II (7), class III (7), class III–IV (1), class IV (1)^231^	↓	↓	NR	—
HCM gene mutations in *MHC7* (16), *TNNT2* (8), or *MYBPC2* (7) (31)^232^	NR	NR	↓	30% reduction in patients with HCM compared with controls; reduction similar in all groups
HHD (NYHA class 0 [10])	=	=	↓	No change in ATP in AS or HHD; 35% decrease in ATP in DCM 28% decrease in PCr in AS, 51% in DCM, no change in HHD 25% decrease in PCr/ATP ratio in HHD
AS (NYHA class II [7], class III [3])	=	↓	NR	
DCM (NYHA class II [1], class III [9])^233^	↓	↓	NR	
AS (10); five followed up^234^	NR	NR	↓	Decrease in PCr/ATP before aortic valve repair
HHD (11)^235^	NR	NR	↓	—
Chronic mitral regurgitation (22)^236^	NR	NR	↓	—
HCM (14)^237^	NR	NR	↓	—
DCM (43 total; 6 restrictive cardiomyopathy, 10 normal systolic and diastolic function; 24 cold preserved from transplantations)^238^	↓	NR	NR	Decrease in ATP in DCM
AI (9; NYHA class average 2.44) or AS (13; NYHA class average 2.77)^239^	NR	NR	↓	Significant reduction in PCr/ATP ratio in patients with AS; trend towards a reduction in patients with AI Significant decrease in PCr/ATP ratio for all patients in NYHA class III, but not those in class I or II
DCM (23; NYHA class average 2.7)^240^	NR	NR	↓	—
AS (41)^241^	↓	↓	NR	—
Severe AS (27)^242^	NR	NR	↓	Hand-grip strength tests (marker of cardiac health) employed in patients
HCM (19)^243^	NR	NR	↓	—
DCM and CHF (NYHA class I [1], class II [3], class III [4])^244^	NR	NR	=	No change with dobutamine infusion
DCM (9), HCM (8)^245^	NR	NR	↓	Decreased PCr/ATP ratio in HCM, but not DCM
CAD (14), DCM (19 total; NYHA class II [4], class III [4], class II–III [7], class III–IV [4])^246^	NR	NR	↓	Decreased PCr/ATP ratio in DCM Trend for decreased PCr/ATP ratio in CAD Relationship exists between severity of HF and decrease in PCr/ATP ratio
DCM (19), ICM (11)^247^	=	NR	NR	No change in ATP levels in DCM biopsies Lower ATP levels in ICM, but not significantly different
Aortic valve disease (6), AI (8)^248^	NR	NR	↓	Decreased PCr/ATP ratio in patients being treated for heart failure
DCM (20)^249^	NR	NR	↓	—
DCM (6), severe LVH (6), mild LVH (5)^250^	NR	NR	=	No change in PCr/ATP ratio in LVH or DCM

AI, aortic insufficiency; AS, aortic stenosis; CAD, coronary artery disease; CHF, congestive heart failure; CK, creatine kinase; DCM, dilated cardiomyopathy; HCM, hypertrophic cardiomyopathy; HFpEF, heart failure with preserved ejection fraction; HHD; hypertensive heart disease; ICM, insertable cardiac monitor; LVH, left ventricular hypertrophy; NR, not reported; PCr, phosphocreatine.
